# The Chlorate-Iodine-Nitrous Acid Clock Reaction

**DOI:** 10.1371/journal.pone.0109899

**Published:** 2014-10-14

**Authors:** Rafaela T. P. Sant'Anna, Roberto B. Faria

**Affiliations:** Instituto de Química, Universidade Federal do Rio de Janeiro, Rio de Janeiro, RJ, Brazil; Brandeis University, United States of America

## Abstract

A new clock reaction based on chlorate, iodine and nitrous acid is presented. The induction period of this new clock reaction decreases when the initial concentrations of chlorate, nitrous acid and perchloric acid increase, but it is independent on the initial iodine concentration. The proposed mechanism is based on the LLKE autocatalytic mechanism for the chlorite-iodide reaction and the initial reaction between chlorate and nitrous acid to produce nitrate and chlorite. This new clock reaction opens the possibility for a new family of oscillating reactions containing chlorate or nitrous acid, which in both cases has not been observed until now.

## Introduction

The discovery of a clock reaction is a very special and rare nonlinear event because it is necessary an autocatalytic sequence of reactions and a short range of reagent concentrations. The first clock reaction containing chlorate (the chlorate-iodine clock reaction), was discovered by our group when studying the kinetic of the chlorate-iodine reaction [1]. This clock reaction is indeed a photochemically induced clock reaction because it needs UV light stimulation to occur [2]. Because UV light can generate small amounts of ozone from dissolved oxygen in water, we investigated the possibility that an ozone solution could substitute for light and trigger the chlorate-iodine clock reaction. In fact, we were able to show that the ozone-iodine-chlorate clock reaction exists and the initial step is the reaction between ozone and iodide [3]. In this same work [3] we have also shown that not all oxidant species, as for example H_2_O_2_, were able to react in such way to produce the autocatalysis which results in the clock reaction behavior.

In the present work we show that nitrous acid can substitute for ozone to produce a new clock reaction which was fully characterized. This new chlorate-iodine-nitrous acid clock reaction opens a new opportunity to find an oscillating system containing chlorate or nitrous acid species, in both cases an unprecedented finding. The possibility to find out an oscillating reaction based on this new clock reaction is greater than in the case of the ozone-iodine-chlorate clock reaction [3] because a nitrous acid solution is much easier to work than the unstable ozone solution.

## Materials and Methods

All reagents were used as received: sodium chlorate (Fluka), perchloric acid (VETEC), resublimed iodine (VETEC), sodium nitrite (Carlo Erba). The solutions were made using conductivity water (18 MΩ) from a Milli-Q Plus system. All experiments were conducted at 25±0.1°C using Hellma Suprasil quartz cuvettes inserted in a jacketed cuvette holder connected to an Etica electronic thermostatic bath.

The clock reaction was followed at 460 nm, which is the maximum of the iodine band, using an Agilent 8453 spectrophotometer in the kinetic mode. Experimental data were acquired at fixed frequency (cycle time) and the data collection time for each point (integration time) was equal to 0.5 s. In these experiments only the tungsten lamp was turned on (deuterium lamp was turned off) to prevent incidence of ultraviolet light on the sample.

All experiments were done at least in triplicate. The experimental curves presented in the figures are typical ones for each set of reagents concentrations. The clock time of these curves does not deviate more than 5% of any other using the same experimental conditions. Similarly, the absorbance values do not spread more than 2% when repeating the same experiment.

The concentration of the iodine (λ_max_ = 460 nm; ε = 740 L mol^−1^ cm^−1^) [4], and nitrous acid (λ_max_ = 358 nm; ε = 52 L mol^−1^ cm^−1^) [5] solutions were measured by spectrophotometry using the indicated bands and molar absorptivity. The nitrous acid solutions were always freshly produced by dissolution of sodium nitrite in perchloric acid aqueous solution to produce the desired nitrous acid and H^+^ concentration.

A semi-implicit Runge-Kutta numerical integration method [6], codified in Turbo Pascal language, was used to produce the simulation results employing the proposed mechanism.

## Results and Discussion

**Figure 1:**
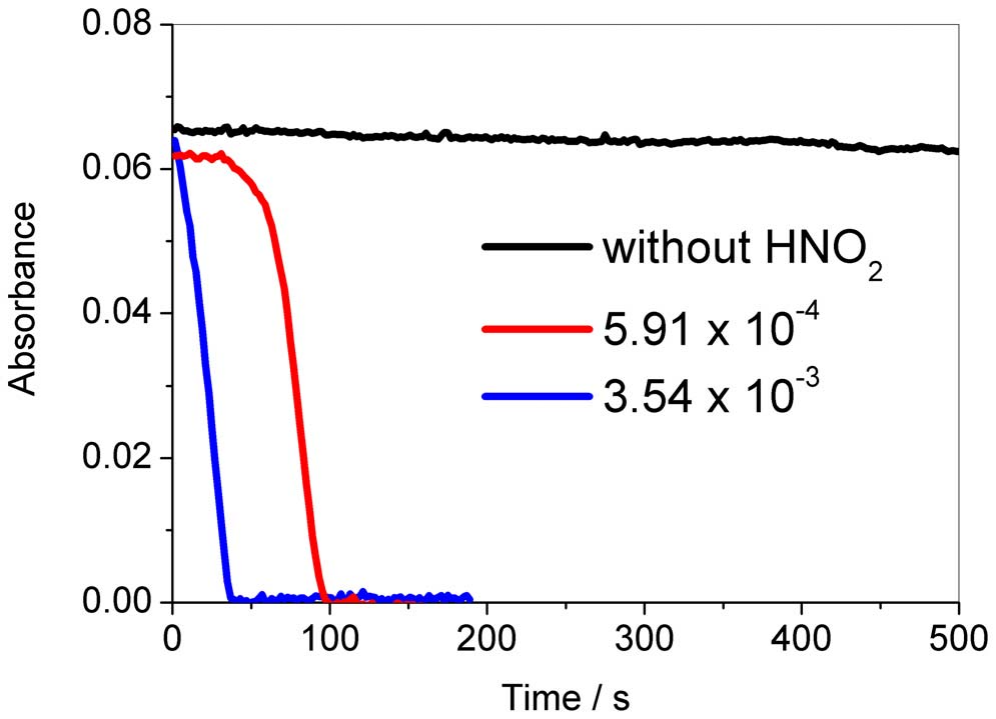
The addition of nitrous acid produces the chlorate-iodine-nitrous acid clock reaction. Following the absorbance at 460 nm for the systems chlorate-iodine-perchloric acid (without addition of HNO_2_) and chlorate-iodine-perchloric acid-nitrous acid (HNO_2_ concentrations indicated in the figure, mol L^−1^), both in the absence of ultraviolet light. Other initial concentrations: [HClO_4_] = 0.948 mol L^−1^; [NaClO_3_] = 0.0251 mol L^−1^; [I_2_] = 8.80×10^−5^mol L^−1^. Experimental data point were measured at every 2 s.

Figure 1 shows that without nitrous acid, no significant change occur at 460 nm during 500 s. However, in the presence of nitrous acid an autocatalytic reaction occurs after a small induction period, which is a typical format for a clock reaction.

**Figure 2:**
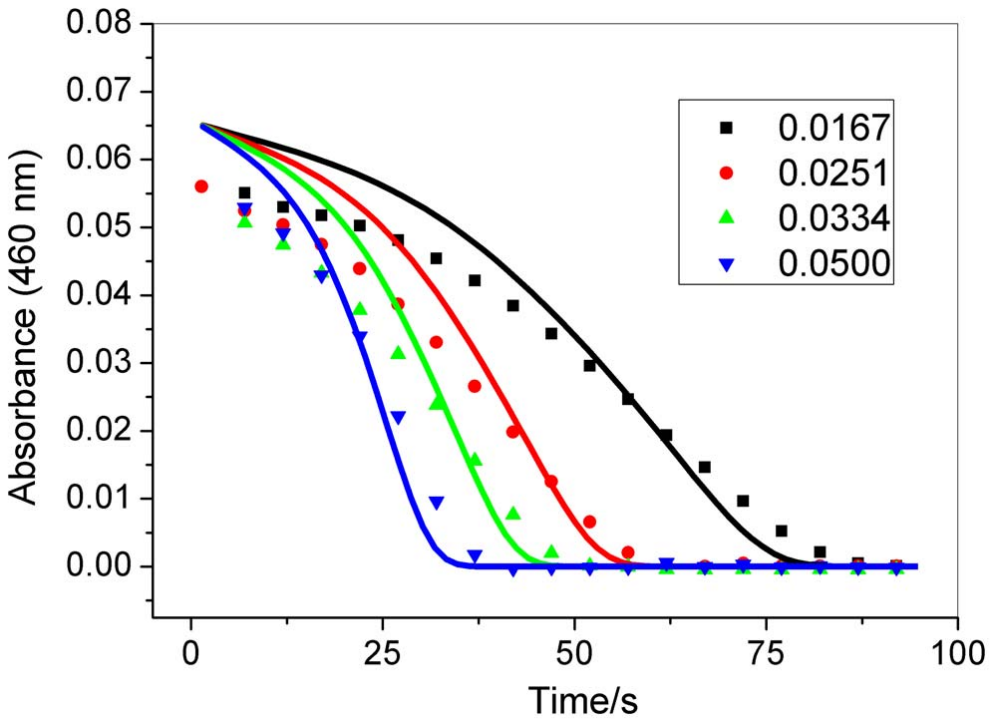
Effect of chlorate concentration (indicated in the figure – mol L^−1^) in the clock time. Other initial concentrations: [HClO_4_] = 0.948 mol L^−1^; [I_2_] = 8.80×10^−5^ mol L^−1^; [HNO_2_] = 1.15×10^−3^mol L^−1^. Experimental (symbols); simulation (continuous lines). Experimental data point were measured at every 5 s.

**Figure 3:**
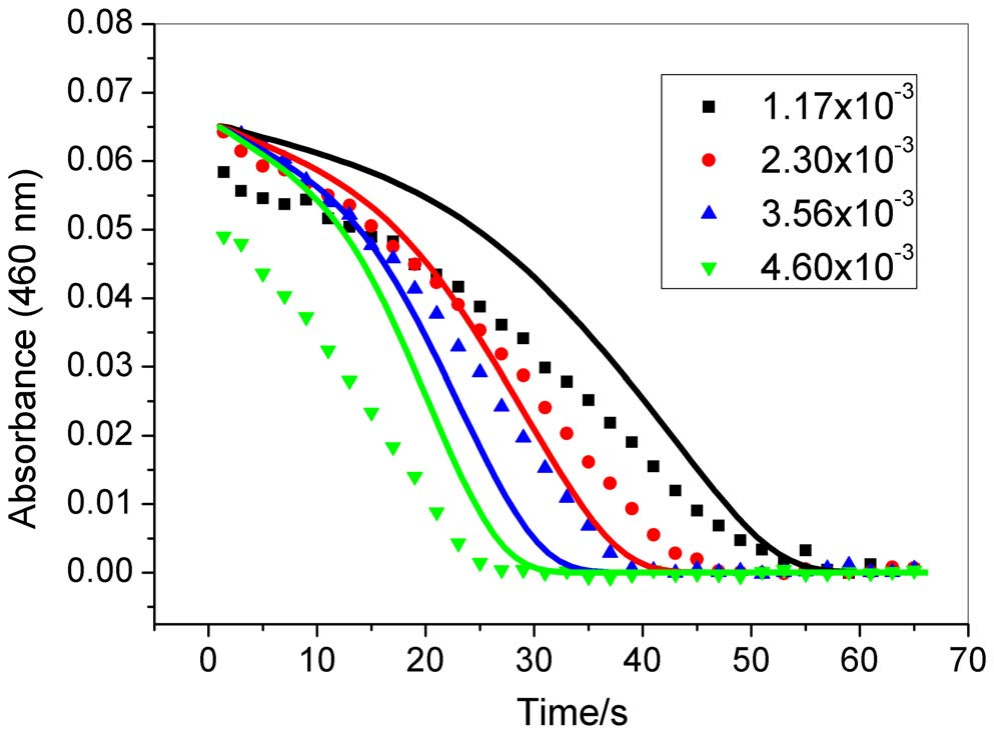
Effect of nitrous acid concentration (indicated in the figure – mol L^−1^) in the clock time. Other initial concentrations: [HClO_4_] = 0.948 mol L^−1^; [NaClO_3_] 0.0251 mol L^−1^; [I_2_] = 8.80×10^−5^ mol L^−1^. Experimental (symbols); simulation (continuous lines). Experimental data point were measured at every 2 s.

**Figure 4:**
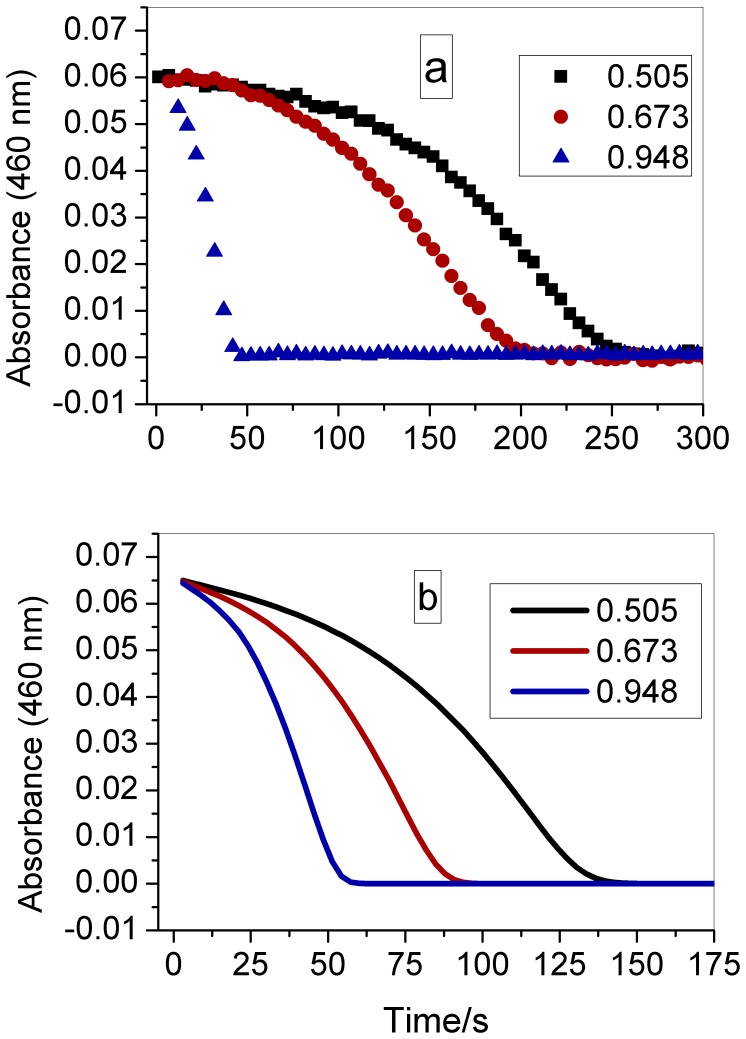
Effect of perchloric acid concentration (indicated in the figure – mol L^−1^) in the clock time. Other initial concentrations: [I_2_] = 8.80×10^−5^ mol L^−1^; [NaClO_3_] = 0.0251 mol L^−1^; [HNO_2_] = 1.15×10^−3^ mol L^−1^. (a) Experimental (symbols); (b) Simulation (continuous lines). Experimental data points were measured at every 5 s.

**Figure 5:**
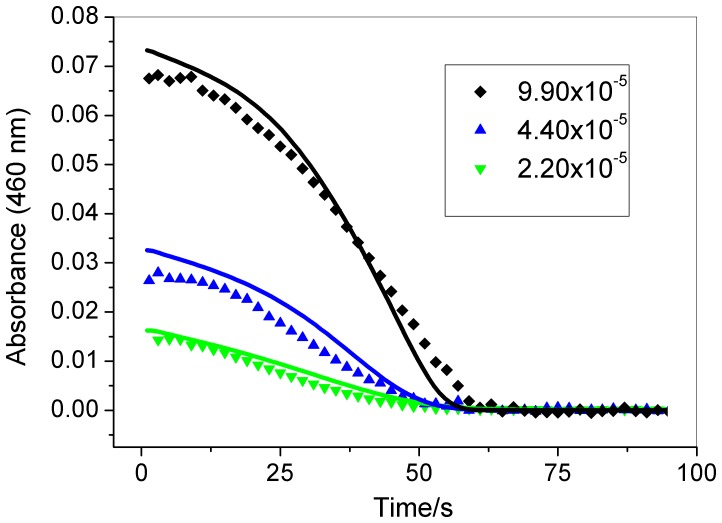
Effect of iodine concentration (indicated in the figure – mol L^−1^) in the clock time. Other initial concentrations: [HClO_4_] = 0.948 mol L^−1^; [NaClO_3_] = 0.0251 mol L^−1^; [HNO_2_] = 1.15×10^−3^ mol L^−1^. Experimental (symbols); simulation (continuous lines). Experimental data point were measured at every 2 s.

Figures 2, 3, and 4 show that the induction period of this chlorate-iodine-nitrous acid clock reaction decreases as the initial concentrations of chlorate, nitrous acid, and perchloric acid are increased. However, the induction period does not change when the initial iodine concentration is modified (Figure 5).

To explain the observed clock behavior we propose the mechanism presented in Table 1, which is based on Reactions 1 to 16, proposed by Lengyel *et al*. [7] to provide the autocatalytic pathway necessary for the clock behavior. Despite of some criticism [8], this mechanism reproduces the chlorite-iodide clock behavior and remains the best available model for this system. To this set of reactions we added the reactions of chlorate with nitrous acid and with HIO_2_, Reactions (17) and (18), respectively.

**Table 1:** Mechanism for the chlorate-iodine-nitrous acid clock reaction.

Number^a^	Reaction	Rate law^b^
1*	I_2_ + H_2_O ↔ HOI + I^−^ + H^+^	1.98×10^−3^ [I_2_]/[H^+^]–3.67×10^9^ [HOI][I^−^]
2*	I_2_ + H_2_O ↔ H_2_OI^+^ + I^−^	5.52×10^−^^2^ [I_2_]–3.48×10^9^ [H_2_OI^+^][I^−^]
3*	HClO_2_ + I^−^ + H^+^ → HOI + HOCl	7.8 [HClO_2_][I^−^]
4**	HClO_2_ + HOI → HIO_2_ + HOCl	6.9×10^7^ [HClO_2_][HOI][H^+^]
5*	HClO_2_ + HIO_2_ → IO_3_^−^ + HOCl + H^+^	1.0×10^6^ [HClO_2_][HIO_2_]
6*	HOCl + I^−^ → HOI + Cl^−^	4.3×10^8^ [HOCl][I^−^]
7*	HOCl + HIO_2_ → IO_3_^−^ + Cl^−^ + 2 H^+^	1.5×10^3^ [HOCl][HIO_2_]
8*	HIO_2_ + I^−^ + H^+^ ↔ 2 HOI	1×10^9^ [HIO_2_][I^−^][H^+^] – 22 [HOI]^2^
9*	2 HIO_2_ → IO_3_^−^ + HOI + H+	25 [HIO_2_]^2^
10*	HIO_2_ + H_2_OI^+^ → IO_3_^−^ + I^−^ + 3 H^+^	110 [HIO_2_][H_2_OI^+^]
11*	Cl_2_ + H_2_O ↔ HOCl + Cl^−^ + H^+^	22 [Cl_2_] – 2.2×10^4^ [HOCl][Cl^−^][H^+^]
12*	Cl_2_ + I_2_ +2 H_2_O → 2 HOI +2 Cl^−^ +2 H^+^	1.5×10^5^ [Cl_2_][I_2_]
13*	Cl_2_ + HOI + H_2_O → HIO_2_ +2 Cl^−^ +2 H^+^	1.0×10^6^ [Cl_2_][HOI]
14*	HClO_2_ ↔ ClO_2_^−^ + H^+^	2×10^8^ [HClO_2_]–1×10^10^ [ClO_2_^−^][H^+^]
15*	HOI + H^+^ ↔ H_2_OI^+^	1×10^10^ [HOI][H^+^]–3.4×10^8^ [H_2_OI^+^]
16*	I_2_ + I^−^ ↔ I_3_^−^	5.6×10^9^ [I_2_][I^−^]–7.5×10^6^ [I_3_^−^]
17	ClO_3_^−^ + HNO_2_ → ClO_2_^−^ + NO_3_^−^ + H^+^	1.8×10^−2^ [ClO_3_^−^][HNO_2_][H^+^]^2^
18	ClO_3_^−^ + HIO_2_ → IO_3_^−^ + HClO_2_	20 [ClO_3_^−^][HIO_2_][H^+^]

a ) * Reactions taken form Lengyel *et al*. [7]. ** Modified from the Lengyel *et al*. [7] including the [H^+^] effect in the rate law.

b ) Rate constants' units are s^−1^, L mol^−1^ s^−1^, L^2^ mol^−2^ s^−1^, and L^3^ mol^−3^ s^−1^ for first-, second-, third-, and forth-order processes, respectively.

Reaction (18) has been used in our model for the chlorate-iodine clock reaction [1] with *k* = 7 L mol^−1^ s^−1^, and in the model for the ozone-iodine-chlorate clock reaction [3] with the same rate constant used here (*k* = 20 L mol^−1^ s^−1^).

Reaction (17) has been studied by Emeish [9] which found the rate law *k*[ClO_3_^−^][HNO_2_][H^+^], *k* = 2.42 L^2^ mol^−2^ s^−1^, at 25°C, using HCl to adjust pH. The use of this rate constant in our model produced a very fast clock event. We were able to simulate the experimental results only after reduce this rate constant to 1.8×10^−2^ L^3^ mol^−3^ s^−1^ (the order of the reaction was changed also; see next paragraph for explanation). Based on the proposed mechanism for the chlorate-chloride reaction [10], it can be considered that the presence of chloride is able to turn chlorate into more reactive species like ClOClO. This may justify the use of a lower rate constant in our system that does not contain HCl.

The qualitative agreement for the acid concentration effect, as shown in Figure 4, was obtained only after the inclusion of one additional H^+^ in the rate laws for Reactions (4), (17) and (18). In the case of Reaction (4), the additional H^+^ intend to adjust the ratios [ClO_2_^−^]/[HClO_2_] and [HOI]/[H_2_OI^+^] which must be affected by changes in [H^+^] as indicated by Jowsa *et al*. [8]. This additional H^+^ in the rate law for Reation (4) has been used before in the model for the ozone-iodine-chlorate clock reaction [3]. For Reaction (18), the additional H^+^ in the rate law can be justified by the same argument, considering the protonation of ClO_3_^−^ and HIO_2_, and has also been used in the model for the ozone-iodine-chlorate clock reaction [3]. In the case of Reaction (17) it was very critical to introduce a square dependence on H^+^ to obtain the qualitative agreement shown in Figure 4. Again, as in our system we do not use HCl, it is possible that the rate law for this reaction has a greater dependence on H^+^ than observed by Emeish [9] in the presence of an excess of chloride.

As can be seen from the simulated results in Figures 2, 3, 4, and 5, the proposed mechanism reproduces the experimental behavior with good agreement for the chlorate, nitrous acid, and iodine effect. In the case of the acid concentration effect, to better show the qualitative agreement between the model and experimental results these are shown using different time scales in Figure 4. The comparison between experimental and simulation results presented in Figure 4 shows that the model reproduces the clock time for [H^+^] = 0,948 M and produces a short clock time for other acid concentrations, presenting a good qualitative agreement in this case.

It is important to notice that the chlorate-iodine-ozone [3] and chlorate-iodine-nitrous acid clock reactions have very different starting reactions. In the first the iodide is oxidized by ozone to HOI which reacts with chlorate producing chlorite which is converted to HClO_2_. In the present work the first step is the chlorate reaction with nitrous acid that produces nitrate and chlorite that goes to HClO_2_. The HClO_2_ then reacts with several iodine species (I-, HOI, and HIO_2_) starting the complex autocatalytic set of reactions. In other words, despite the fact that we have substituted ozone for nitrous acid, this species is oxidized by chlorate while ozone is reduced by iodide. It means that this new chlorate-iodine-nitrous acid clock reaction can not be considered a simple and obvious variation of the chlorate-iodine-light clock reaction [1], [2] or of the chlorate-iodine-ozone clock reaction [3].

These results above show that a new clock reaction involving chlorate, iodine and nitrous acid has been discovered and was fully characterized. It is the third clock reaction involving chlorate and it is an important step toward the discovery of an oscillating reaction involving chlorate, because the nitrous acid aqueous solution is much easier to handle than the ozone aqueous solution employed in the ozone-iodine-chlorate system [3]. Using this clock reaction and the cross-shape diagram method [11] should allow to find the experimental conditions to observe the first oscillating reaction involving chlorate.

## Supporting Information

File S1 **Absorbance experimental data values for all curves shown in the figures.**
(DOC)
