# Supplementary material for: The Chlorate-Iodine-Nitrous Acid Clock Reaction
Source: PLoS One. 2014 Oct 14;9(10):e109899. doi: 10.1371/journal.pone.0109899 (PMC4196969; doi:10.1371/journal.pone.0109899)
Supplement: File S1 — Absorbance experimental data values for all curves shown in the figures. (DOC) [file pone.0109899.s001.doc]

Supporting Information

The sequence of data below contain the absorbance values for each experimental curve presented in Figures 1 to 5. As the data were obtained at a fixed frequency, only the time for the first experimental points and the frequency is specified.

FIGURE 1

Color of the curve: black

Label of the curve: without HNO_2_

First experimental data point at 1.4 s

Second experimental data point at 3 s

All other experimental data points spaced by 2 seconds (5 s, 7 s, 9 s, … 499 s)

Absorbance values:

0.06545, 0.06584, 0.06573, 0.06529, 0.06533, 0.06513, 0.06502, 0.0652, 0.06528, 0.06509, 0.06536, 0.0652, 0.06525, 0.06503, 0.06537, 0.06521, 0.0657, 0.0658, 0.06485, 0.06508, 0.06515, 0.06523, 0.06571, 0.06496, 0.06529, 0.06527, 0.06572, 0.06539, 0.06534, 0.06521, 0.06524, 0.06511, 0.06519, 0.06534, 0.06512, 0.06527, 0.06506, 0.06501, 0.06508, 0.06519, 0.06495, 0.06499, 0.06511, 0.0651, 0.06495, 0.06505, 0.06524, 0.06519, 0.06477, 0.06469, 0.06481, 0.06483, 0.06474, 0.06478, 0.06477, 0.06468, 0.06443, 0.06478, 0.06449, 0.06459, 0.06428, 0.06435, 0.06437, 0.06439, 0.06451, 0.06448, 0.06444, 0.06467, 0.06452, 0.06466, 0.06457, 0.06434, 0.06448, 0.06437, 0.06453, 0.0647, 0.06449, 0.06439, 0.06448, 0.06449, 0.06498, 0.06439, 0.06426, 0.06424, 0.06412, 0.06467, 0.06506, 0.06504, 0.06425, 0.06439, 0.06436, 0.06417, 0.06421, 0.06438, 0.06446, 0.06431, 0.06441, 0.06434, 0.06429, 0.06448, 0.06424, 0.06406, 0.06435, 0.06424, 0.06448, 0.06421, 0.06438, 0.06402, 0.06432, 0.06412, 0.06416, 0.06404, 0.06409, 0.06411, 0.06425, 0.06406, 0.06397, 0.06428, 0.06417, 0.06386, 0.06418, 0.06395, 0.06406, 0.06399, 0.06408, 0.06384, 0.06383, 0.06379, 0.06381, 0.06412, 0.06406, 0.06391, 0.06362, 0.06371, 0.06368, 0.06376, 0.06382, 0.06475, 0.06391, 0.06379, 0.06389, 0.06373, 0.06371, 0.06403, 0.06366, 0.06398, 0.06407, 0.06411, 0.0634, 0.06355, 0.06373, 0.06367, 0.0638, 0.06369, 0.06358, 0.06366, 0.06376, 0.0636, 0.06344, 0.06352, 0.06361, 0.06362, 0.0638, 0.06376, 0.06381, 0.06386, 0.06364, 0.06379, 0.06391, 0.06395, 0.0641, 0.06373, 0.06374, 0.06373, 0.06388, 0.0639, 0.06371, 0.06372, 0.06399, 0.06386, 0.06409, 0.06377, 0.06367, 0.0639, 0.06384, 0.06395, 0.06385, 0.06395, 0.06401, 0.06409, 0.06413, 0.06405, 0.06404, 0.06391, 0.06375, 0.06377, 0.06372, 0.06383, 0.06368, 0.06395, 0.06371, 0.06368, 0.06363, 0.06378, 0.06363, 0.06348, 0.06367, 0.06359, 0.06328, 0.06377, 0.06359, 0.06306, 0.06315, 0.06304, 0.06301, 0.06295, 0.06294, 0.06308, 0.06324, 0.06306, 0.06282, 0.06278, 0.06267, 0.06269, 0.06243, 0.06256, 0.06268, 0.06275, 0.06285, 0.0628, 0.0627, 0.06283, 0.06269, 0.06292, 0.06268, 0.06285, 0.06283, 0.06305, 0.06305, 0.06292, 0.06291, 0.06287, 0.06282, 0.0628, 0.06292, 0.06266, 0.06243, 0.06248, 0.06252, 0.06239

Color of the curve: red

Label of the curve: 5.91 × 10^-4^

First experimental data point at 1.4 s

Second experimental data point at 3 s

All other experimental data points spaced by 2 seconds (5 s, 7 s, 9 s, … 47 s)

Absorbance values:

0.06181, 0.06181, 0.06196, 0.06177, 0.0619, 0.0622, 0.06186, 0.0614, 0.06138, 0.06181, 0.06183, 0.06161, 0.06129, 0.06166, 0.06176, 0.06211, 0.06125, 0.06127, 0.06086, 0.06012, 0.05995, 0.05966, 0.05912, 0.05864, 0.05829, 0.0577, 0.05677, 0.05646, 0.05564, 0.0551, 0.05344, 0.05218, 0.05002, 0.04777, 0.04572, 0.04343, 0.0401, 0.03617, 0.03234, 0.02865, 0.02468, 0.02058, 0.01655, 0.01315, 0.00926, 0.00626, 0.00348, 0.00174, 3.9E-4, 3E-5, -4.6E-4, -3.1E-4, 0, -7.3E-4, 9E-5

Color of the curve: blue

Label of the curve: 3.54 × 10^-3^

First experimental data point at 1.4 s

Second experimental data point at 3 s

All other experimental data points spaced by 2 seconds (5 s, 7 s, 9 s, … 109 s)

Absorbance values:

0.06395, 0.06256, 0.06034, 0.05719, 0.054, 0.05215, 0.04775, 0.04581, 0.04137, 0.03769, 0.03292, 0.02919, 0.02421, 0.01967, 0.01527, 0.01092, 0.00685, 0.00288, 6.09673E-4, 3.90327E-4, 1.0288E-5, 5.33379E-4, 8.9443E-5, 2.54429E-4

FIGURE 2

Symbol: square

Color of the symbol: back

Label of the curve: 0.0167

First experimental data point at 7 s

Second experimental data point at 12 s

All other experimental data points spaced by 5 seconds (17 s, 22 s, 27 s, … 102 s)

Absorbance values:

0.05508, 0.05299, 0.05178, 0.05027, 0.04811, 0.04539, 0.04215, , 0.03842, 0.03436, 0.02958, 0.02465, 0.01937, 0.01466, 0.00964, 0.00524, 0.00215, 5.1E-4, 1E-4, 7E-5, 8E-5

Symbol: circle

Color of the symbol: red

Label of the curve: 0.0251

First experimental data point at 1.4 s

Second experimental data point at 7 s

All other experimental data points spaced by 5 seconds (12 s, 17 s, 22 s, 27 s, … 82 s)

Absorbance values:

0.05601, 0.05245, 0.05038, 0.04749, 0.04392, 0.03868, 0.03307, 0.02658, 0.01984, 0.01255, 0.00661, 0.00208, 2.1E-4, 3E-5, 4.7E-4, 7E-5, 7E-5

Symbol: triangle pointing up

Color of the symbol: green

Label of the curve: 0.0334

First experimental data point at 7 s

Second experimental data point at 12 s

All other experimental data points spaced by 5 seconds (17 s, 22 s, 27 s, … 67 s)

Absorbance values:

0.05067, 0.04745, 0.04328, 0.03779, 0.03131, 0.0238, 0.01561, 0.00765, 0.002, 1.2E-4, 0,

-3.7E-4, -4.3E-4

Symbol: triangle pointing down

Color of the symbol: blue

Label of the curve: 0.0500

First experimental data point at 7 s

Second experimental data point at 12 s

All other experimental data points spaced by 5 seconds (17 s, 22 s, 27 s, … 62 s)

Absorbance values:

0.05295, 0.04916, 0.04297, 0.03397, 0.02218, 0.00963, 0.00176, -2.5E-4, -1.2E-4, -1.1E-4,

-6E-5, 5.7E-4

FIGURE 3

Symbol: square

Color of the symbol: back

Label of the curve: 1.17 × 10^-3^

First experimental data point at 1.4 s

Second experimental data point at 3 s

All other experimental data points spaced by 2 seconds (5 s, 7 s, 9 s, … 65 s)

Absorbance values:

0.05838, 0.05564, 0.0546, 0.05371, 0.05439, 0.0516, 0.05041, 0.04892, 0.04826, 0.0449, 0.04343, 0.04168, 0.03877, 0.03612, 0.03414, 0.02985, 0.02781, 0.02517, 0.02185, 0.01906, 0.0155, 0.01197, 0.00908, 0.00688, 0.00471, 0.00335, 0.00144, 0.00324, 4.34875E-4,

-3.91006E-5, 0.00123, 2.58446E-4, 4.83513E-4

Symbol: circle

Color of the symbol: red

Label of the curve: 2.30 × 10^-3^

First experimental data point at 1.4 s

Second experimental data point at 3 s

All other experimental data points spaced by 2 seconds (5 s, 7 s, 9 s, 11 s, … 55 s)

Absorbance values:

0.06426, 0.06148, 0.05929, 0.0587, 0.05673, 0.05501, 0.05356, 0.05051, 0.04756, 0.04492, 0.04229, 0.03905, 0.03532, 0.03183, 0.02875, 0.02408, 0.02035, 0.01613, 0.01304, 0.00932, 0.00551, 0.00284, 0.00197, 2.5E-4, 9E-5, 1E-5, -1.2E-4, 5.2E-4

Symbol: triangle pointing up

Color of the symbol: blue

Label of the curve: 3.56 × 10^-3^

First experimental data point at 3 s

Second experimental data point at 5 s

All other experimental data points spaced by 2 seconds (7 s, 9 s, 11 s, … 45 s)

Absorbance values:

0.06395, 0.06256, 0.06034, 0.05719, 0.054, 0.05215, 0.04775, 0.04581, 0.04137, 0.03769, 0.03292, 0.02919, 0.02421, 0.01967, 0.01527, 0.01092, 0.00685, 0.00288, 6.09673E-4, 3.90327E-4, 1.0288E-5, 5.33379E-4

Symbol: triangle pointing down

Color of the symbol: green

Label of the curve: 4.60 × 10^-3^

First experimental data point at 1.4 s

Second experimental data point at 3 s

All other experimental data points spaced by 2 seconds (5 s, 7 s, 9 s, 11 s, … 35 s)

Absorbance values:

0.04901, 0.04795, 0.0436, 0.04032, 0.03723, 0.03239, 0.02802, 0.02338, 0.01835, 0.01403, 0.00884, 0.00428, 0.00145, 4E-4, 4.4E-4, 0, 2.6E-4, -6.1E-4

FIGURE 4

Symbol: square

Color of the symbol: back

Label of the curve: 0.505

First experimental data point at 1.4 s

Second experimental data point at 7 s

All other experimental data points spaced by 5 seconds (12 s, 17 s, 22 s, … 282 s)

Absorbance values:

0.06007, 0.06037, 0.05951, 0.05949, 0.05931, 0.0581, 0.0585, 0.05829, 0.05836, 0.05791, 0.05724, 0.05724, 0.05638, 0.05595, 0.05574, 0.0564, 0.05483, 0.05376, 0.05341, 0.0536, 0.05244, 0.05256, 0.05072, 0.05068, 0.04904, 0.04867, 0.04665, 0.04567, 0.04486, 0.04398, 0.043, 0.04101, 0.03861, 0.03745, 0.03564, 0.03356, 0.03186, 0.02964, 0.0264, 0.02508, 0.02174, 0.0204, 0.01663, 0.0145, 0.01247, 0.00937, 0.00739, 0.00562, 0.0039, 0.00242, 0.00177, 0.00108, 7.74933E-4, 6.20438E-4, 3.18123E-4, 0, -1.8E-4

Symbol: circle

Color of the symbol: red

Label of the curve: 0.673

First experimental data point at 7 s

Second experimental data point at 12 s

All other experimental data points spaced by 5 seconds (17 s, 22 s, 27 s, … 227 s)

Absorbance values:

0.05915, 0.05941, 0.06045, 0.05942, 0.0592, 0.0598, 0.05873, 0.05833, 0.05718, 0.05619, 0.05606, 0.05515, 0.05398, 0.05296, 0.05159, 0.05048, 0.04964, 0.04794, 0.04664, 0.0449, 0.04364, 0.04148, 0.03923, 0.03698, 0.03574, 0.03323, 0.03045, 0.02824, 0.02525, 0.02316, 0.0207, 0.01746, 0.01488, 0.0123, 0.01058, 0.00693, 0.00504, 0.00359, 0.00194, 0.00158, 8.3E-4, 8.4E-4, -1.6E-4, 0.00108, -3.4E-4

Symbol: triangle pointing up

Color of the symbol: blue

Label of the curve: 0.948

First experimental data point at 12 s

Second experimental data point at 17 s

All other experimental data points spaced by 5 seconds (22 s, 27 s, 32 s, … 62 s)

Absorbance values:

0.05345, 0.04966, 0.04347, 0.03447, 0.02268, 0.01013, 0.00226, 2.5E-4, 3.8E-4, 3.9E-4, 4.4E-4

FIGURE 5

Symbol: square

Color of the symbol: back

Label of the curve: 9.90 × 10^-5^

First experimental data point at 1.4 s

Second experimental data point at 3 s

All other experimental data points spaced by 2 seconds (5 s, 7 s, 9 s, … 75 s)

Absorbance values:

0.06751, 0.0682, 0.06699, 0.06762, 0.06787, 0.06504, 0.06403, 0.06325, 0.06157, 0.05919, 0.05744, 0.05602, 0.05368, 0.05198, 0.04919, 0.0464, 0.04386, 0.04082, 0.03732, 0.03415, 0.03097, 0.02741, 0.02419, 0.02034, 0.01752, 0.01358, 0.00981, 0.00823, 0.00499, 0.00188, 5.3E-4, 0.00118, -2.5E-4, 6.5E-4, -1E-4, -4.5E-4, -4E-4, -4.7E-4

Symbol: circle

Color of the symbol: red

Label of the curve: 4.40 × 10^-5^

First experimental data point at 1.4 s

Second experimental data point at 3 s

All other experimental data points spaced by 2 seconds (5 s, 7 s, 9 s, … 75 s)

Absorbance values:

0.02637, 0.02799, 0.02678, 0.02671, 0.02653, 0.02604, 0.02536, 0.02463, 0.0234, 0.02259, 0.02089, 0.01901, 0.01772, 0.01611, 0.01478, 0.01323, 0.01172, 0.01021, 0.00875, 0.00758, 0.00618, 0.00552, 0.00401, 0.00277, 0.00241, 0.00132, 0.00137, 8.6E-4, 0.00186, 1.0693E-5, 3.25405E-4, 2.267E-4, -1.07563E-4, -6.95E-6, 8.3172E-5, -2.14375E-4, 4.77516E-4, 5.9E-4

Symbol: triangle pointing up

Color of the symbol: blue

Label of the curve: 2.20 × 10^-5^

First experimental data point at 3 s

Second experimental data point at 5 s

All other experimental data points spaced by 5 seconds (7 s, 9 s, 1 s, … 75 s)

Absorbance values:

0.0143, 0.01447, 0.01443, 0.0133, 0.01308, 0.01233, 0.01169, 0.0108, 0.01011, 0.00922, 0.00843, 0.00761, 0.00688, 0.00605, 0.00533, 0.0046, 0.00407, 0.00339, 0.00288, 0.00235, 0.00181, 0.00166, 0.00112, 7.91619E-4, 7.73022E-4, 3.66757E-4, 3.29087E-4, 3.62466E-4, 2.65191E-4, 1.1451E-4, 2.51363E-4, 2.03679E-4, 1.14987E-4, 1.50273E-4, 1.78883E-4, 1.86036E-4, 1.7236E-5
